# Monomelic amyotrophy with proximal upper limb involvement: a case report

**DOI:** 10.1186/s13256-016-0843-5

**Published:** 2016-03-17

**Authors:** Eman Al-Ghawi, Talal Al-Harbi, Adnan Al-Sarawi, Mohamed Binfalah

**Affiliations:** 1 Ministry of Health, Building 1228, Road 4025, Juffair, 340 Kingdom of Bahrain; 2 Department of Neurology, King Fahad Specialist Hospital-Dammam, 6830 Ammar bin Thabit St, Al Muraikabat, Dammam, 32253-3202 Saudi Arabia; 3 University Medical Center, King Abdullah Medical City, P.O. Box 26671, Adliya, Kingdom of Bahrain

**Keywords:** Amyotrophic lateral sclerosis, Electromyography, Hirayama disease, Magnetic resonance imaging, Proximal monomelic amyotrophy

## Abstract

**Background:**

Monomelic amyotrophy is an uncommon, benign, unilateral disorder of the lower motor neurons, affecting predominantly the hand and forearm muscles. Proximal involvement of the arm and shoulder muscles is an unusual presentation that has been rarely reported in the literature.

**Case presentation:**

A 28-year-old white man presented with insidious-onset, slowly progressive, unilateral weakness and atrophy of his left shoulder girdle and deltoid muscles. A neurological examination revealed weakness and atrophy in his left deltoid, infraspinatus and supraspinatus muscles. Electromyography demonstrated an active and chronic neurogenic pattern affecting his left C5 and C6 myotomes; magnetic resonance imaging of his cervical spine was normal. He did well with conservative treatment.

**Conclusions:**

Upper limb proximal form of monomelic amyotrophy is a rare clinical entity with a wide differential diagnosis. Physicians, especially neurologists, should be familiar with this benign condition to avoid inappropriately labeling patients as having amyotrophic lateral sclerosis and other disorders with less favorable outcomes.

## Background

Monomelic amyotrophy (MA), also known as Hirayama disease, is a rare, benign lower motor neuron disease. Hirayama *et al*. originally reported this clinical entity in 1959, and called it “juvenile muscular atrophy of unilateral upper extremity” [1]. This disease is characterized by muscle wasting and weakness, affecting predominantly the lower cervical myotomes [2]. It affects mostly young males in their teens and twenties. The disease is more prevalent in India, Japan, and other Asian countries, but many cases have been reported from other parts of the world as well [3–5]. One report from India found that MA comprises approximately 12.8 % of lower motor neuron diseases [5].

We report the case of a patient with MA, who presented with symptoms and signs in his proximal upper limb, a location rarely described in this disease [5–7].

## Case presentation

A 28-year-old, right-handed white man presented with insidious-onset, slowly progressive weakness and wasting of his left deltoid and shoulder girdle muscles for the last 5 years. Initially, he noticed difficulty with abducting his left arm and raising it above head level. The weakness progressed slowly over the first 3 years of initial symptoms, and then became static. He denied history of trauma, shoulder pain, or sensory symptoms, and he was not on any medications prior to development of symptoms.

**Fig. 1:**
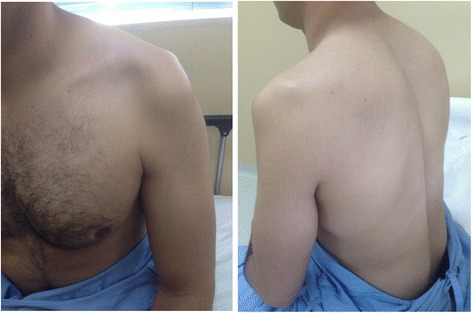
Left shoulder photograph. Atrophy of the deltoid, supraspinatus and infraspinatus muscles

A neurological examination demonstrated significant atrophy of his left deltoid, supraspinatus and infraspinatus muscles, with no fasciculations (Fig. 1). His muscle strength, (according to Medical Research Council) was 3 in his deltoid, supraspinatus and infraspinatus muscles, 4+ in his biceps and teres major, and 5 in other muscles. Deep tendon reflexes and sensory examination were normal. There were no upper motor neuron signs.

Nerve conduction studies (NCS) of his left upper limb were normal. Electromyography (EMG) revealed fasciculations and fibrillation potentials in his biceps, deltoid and supraspinatus. Large, polyphasic motor unit action potentials with prolonged duration and reduced recruitment pattern were seen in the same muscles; the infraspinatus could not be tested due to severe degree of atrophy. EMG of other muscles in his upper and lower extremities, as well as cervical paraspinal muscles, was normal.

**Fig. 2:**
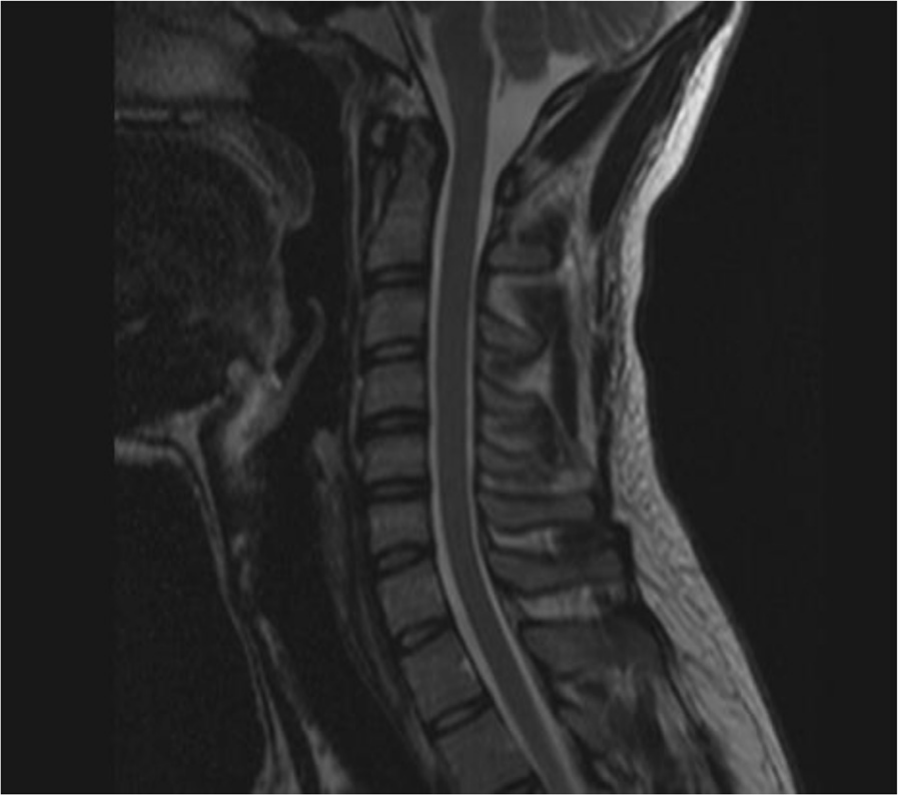
Cervical spine magnetic resonance imaging (1.5 Tesla). Sagittal T2 section, demonstrating normal spinal cord and dural canal in the neutral position

Blood investigations, including complete blood count, sedimentation rate, renal, liver and thyroid function tests, creatine kinase (CK), and vitamins. B12 and D3 were normal. There were negative results for vasculitis screening (rheumatoid factor, antinuclear antibody, extractable nuclear antigens, antiphospholipid antibody) and viral serology: human immunodeficiency virus (HIV), hepatitis B and C. Magnetic resonance imaging (MRI) of his cervical spine, done in the neutral position, was normal (Fig. 2), but a flexion-position MRI was not done.

He was diagnosed to have an upper limb proximal form of benign MA. He was prescribed a regular physiotherapy program to strengthen his shoulder muscles and improve range of movement. His muscle strength and functions have remained stable over the past 2 years. He is currently working as a clerk, with no functional limitations due to his illness. He is doing regular exercises given by his physiotherapist on a daily basis.

## Discussion

MA, or Hirayama disease, is a rare benign disorder of the lower motor neurons. This condition was initially reported by Hirayama *et al*. in 1959 [1]; they called it “juvenile muscular atrophy of unilateral upper extremity”. The disease is more prevalent in India, Japan, and other Asian countries, but many similar cases have been reported from other parts of the world as well [3–5]. For instance, one report from India found that MA comprises approximately 12.8 % of lower motor neuron diseases [5]. To the best of our knowledge, this is the first case reported from our region (Arabian Gulf).

MA is characterized by insidious-onset, asymmetric, unilateral weakness and atrophy of the hand and forearm muscles, with sparing of the brachioradialis, giving rise to an appearance called ‘oblique amyotrophy’ [1, 8, 9]. Several case series have described predominant lower limb involvement [4, 10].

MA typically affects males between the ages of 15 and 25 years [9], but it can occur in females as well [4, 11]. The disease progresses slowly over several years, before reaching a stationary stage [3, 9]. Bilateral, usually asymmetric, but also symmetric, ‘bimelic’ forms affecting the upper limbs have been observed [3].

Our patient presented with an uncommon form of MA, affecting the shoulder and arm muscles instead of the commonly seen hand and forearm disease. Rare cases of proximal upper limb MA have been reported in the literature [4–7]. De Freitas and Nascimento [4] studied 21 cases of MA involving the upper or lower extremities. Only one patient had proximal unilateral upper limb involvement; his MRI cervical spine and NCS were normal, while EMG revealed denervation in the affected muscles, similar to our patient. Another study [2] described 102 patients with MA. Only five of them had predominantly unilateral shoulder girdle weakness; however, EMG showed evidence of denervation not only in C5 and 6 myotomes, but also C8 and T1 muscles, albeit to a lesser degree, denoting a more extensive disease, which was not seen in our patient.

Our patient did not have sensory symptoms or upper motor neuron signs during 5 years, or clinical or electrophysiological evidence for a widespread disease in bulbar or other limb muscles, indicating the benign nature of this condition.

Other characteristic clinical findings compatible with MA include course irregular tremor (polyminimyoclonus) of the fingers in affected hand, exaggeration of weakness on exposure to cold (cold paresis), and absence of sensory loss, pyramidal, cerebellar, cranial nerves, and sphincter deficits [9, 10, 12].

The differential diagnosis of MA includes the distal form of spinal muscular atrophy, amyotrophic lateral sclerosis (ALS), post-polio syndrome, multifocal motor neuropathy with conduction block (MMNCB), as well as structural lesions of the cervical cord. These clinical entities can be identified by specific clinical, radiological and electrophysiological features.

The pathophysiology of MA remains unknown [4], but several postulations have been considered, such as viral infections, ischemia to anterior horn cells, and atopy [6, 9]. In 1987, Kikuchi *et al*. first proposed that a tight dural canal may be an underlying predisposing factor [13]. Hirayama [9] suggested a model of focal venous ischemia due to compression and flattening of the lower cervical cord arising from forward displacement of the cervical dural sac and spinal cord, caused by recurrent neck flexion. 

MRI findings in MA are conflicting, with some studies showing localized asymmetric spinal cord atrophy, prominence and enhancement of posterior epidural venous plexus, and anterior shifting of posterior dural sac on flexion [14]. In one case series, more than 90 % of patients were found to have several characteristic MRI abnormalities on flexion study, such as loss of dural attachment, anterior displacement of dorsal dura, and enhancing epidural crescent [12]. Other studies have demonstrated no MRI differences in the anterior-posterior cord diameter between patients and controls [9, 15].

An autopsy study on a patient with MA noticed anterior horn cell shrinkage and necrosis, various degrees of degeneration of small and large nerve cells, mild gliosis, and some circulatory insufficiency in the territory of the spinal cord. These abnormalities extended from C5 to T1 levels but were more marked at C7 and C8 [10].

Electrophysiological studies (NCS and EMG) are an essential part of the diagnostic process, showing mostly chronic denervation changes in affected muscles, with or without acute denervation potentials (fasciculations, positive sharp waves and fibrillations potentials). These studies are also helpful in excluding other apparently similar conditions (e.g. ALS, MMNCB, brachial plexopathy). Milder denervation changes can be seen in clinically unaffected muscles as well [4].

There is no consensus on the treatment of MA, but the use of cervical collar therapy during the acute progressive stage of the disease has been advocated [12]. The role of surgery in patients who progress despite conservative treatment is controversial, but duraplasty, anterior cervical decompression, and reconstruction with tendon transfers have yielded encouraging results in some patients [11]. Physiotherapy is helpful in preventing complications resulting from immobility such as joint stiffness and muscle wasting.

## Conclusions

MA should be suspected in patients presenting with slowly progressive weakness and atrophy restricted to one limb, followed by a static phase. While most reported cases involve the lower cervical myotomes, affecting the hand and forearm muscles, proximal upper limb involvement can be seen rarely. EMG and MRI studies are helpful in confirming the diagnosis and ruling out other clinical entities presenting in a similar fashion. Treatment is conservative in most patients, with the use of a cervical collar in appropriate cases, and physiotherapy.

## Consent

Written informed consent was obtained from the patient for publication of this case report and accompanying images. A copy of the written consent is available for review by the Editor-in-Chief of the journal.

## Abbreviations

ALS: Amyotrophic lateral sclerosis
CK: Creatine kinase
EMG: Electromyography
HIV: Human immunodeficiency virus
MA: Monomelic amyotrophy
MMNCB: Multifocal motor neuropathy with conduction block
MRI: Magnetic resonance imaging
NCS: Nerve conduction studies
